# Molecular simulation study on competitive adsorption mechanism of gas power generation tail gas in coal micropore structure

**DOI:** 10.1038/s41598-026-49416-z

**Published:** 2026-04-27

**Authors:** Ge Huang, Zijuan Zhu, Fengwei Dai, Xun Zhang, Gang Bai, Yilong Zhang

**Affiliations:** 1 College of Safety Science and Engineering, Liaoning Technical University, Huludao, 125105 Liaoning China; 2 Key Laboratory of Mine Thermodynamic Disaster & Control of Ministry of Education, Liaoning Technical University, Huludao, 125105 Liaoning China; 3 College of Mining Engineering, Liaoning Technical University, Fuxin, 123000 Liaoning China

**Keywords:** Gas power generation tail gas, Fire prevention, CO_2_ storage, Coal micropores, Competitive adsorption, Energy science and technology, Engineering, Environmental sciences

## Abstract

To study the effect of coal micropore structure on the adsorption behavior of gas-fired power tail gas, low-temperature N_2_ adsorption, low-pressure CO_2_ adsorption, and molecular simulation were combined to analyze the competitive adsorption characteristics of tail-gas components in the micropores of Hongqingliang bituminous coal and the pore-size effect. The results show that the single-component adsorption capacity follows CO_2_ > O_2_ > N_2_, with the average CO_2_ adsorption being 2.12 mmol/g, about 1.5 and 1.7 times that of O_2_ and N_2_, respectively, and the same order for adsorption heat. In multicomponent competitive adsorption, CO_2_ still dominates; although O_2_ reduces CO_2_ adsorption by about 2.02%, the synergistic effect among tail-gas components increases the total adsorption by 5.74% compared with the N_2_–CO_2_ system, by 21.43% compared with the pure N_2_ system, and by 33.13% compared with a low-CO_2_ atmosphere. Pore-size analysis indicates that CO_2_ shows the strongest competitive advantage and selectivity in ultra-micropores, and this advantage gradually decreases with increasing pore size. These findings highlight the key role of ultra-micropore structure in enhancing adsorption competitiveness and energy differences, providing a theoretical basis for tail-gas resource utilization and CO_2_ sequestration.

## Introduction

The goaf formed after coal mining provides a good geological environment for carbon dioxide storage and is also a potential place for comprehensive gas recovery and utilization^1^. Under the dual goals of reducing carbon emissions and improving resource efficiency^2^, how to realize the safe long-term storage of CO_2_ in the goaf while realizing the effective reuse of gas components in the flue gas of the power plant has become an urgent problem to be solved^3^.

In recent years, extensive research around the world has focused on the utilization and management of exhaust gas from gas power generation^4^. Wu S^5^, Liu Z^6^ et al. proposed to inject waste gas directly into underground coal seams to achieve local storage of CO_2_. This method can not only make CO_2_ closed for a long time, thus reducing atmospheric accumulation, but also inhibit the oxidation and spontaneous combustion of residual coal. In the field of enhanced coalbed methane recovery (ECBM)^7^, UNAL^8^ and other researchers used Australian bituminous coal to investigate the gas adsorption behavior, and found that the adsorption capacity of N_2_ and O_2_ was significantly weaker than that of CH_4_ and CO_2_. Syed A^9^, Noely T T^10^, Xiang J H^11^ further explored the gas injection technology to improve the recovery of methane. Bo Cong L^12^ and Wen Z^13^ systematically studied the competitive adsorption behavior of multi-component gases in the process of CO_2_ flooding CH_4_, and pointed out that CO_2_ is superior to methane in the adsorption process. Long W^14^ and Levy J H^15^ investigated the CO_2_ displacement mechanism under different gas injection modes in tectonic coal. Li Z^16,17^ found that the injection of mixed gas containing CO_2_ can more effectively improve the adsorption and replacement efficiency of coal to gas than single nitrogen injection, and inhibit the spontaneous combustion tendency of coal while storing a large amount of CO_2_.

On the other hand, coal, as a natural porous adsorbent, has a multi-level pore system from nano-scale micropores to micron-scale mesopores and macropores^18^. Zhou S^19^ et al. reported that micropores are dominant in coalbed methane reservoirs, and their wide internal surface area has a decisive contribution to the overall adsorption performance of coal. Zhang J^20^ et al. further proved that pores smaller than 10 nm were the main sites for CH_4_ adsorption and desorption. Among them, the pore classification method proposed by Hodot B.B. (1966) is widely accepted because it fully considers the flow characteristics of gas in different pore structures. The pores in coal can be divided into micropores (<10 nm), transition pores (10–100 nm), mesopores (100–1000 nm) and macropores (>1000 nm).

In recent years, molecular simulation has become an important method to study the gas adsorption behavior in coal. Van Megen W^21^, Evans R^22^ and others used grand canonical Monte Carlo and density functional theory methods to study the adsorption characteristics in various pore structures. Cracknell R F^23^ et al. used molecular simulation to study the selectivity of CH_4_, CO_2_ and N_2_ in slit-type channels. Wang J^24^ proved that there is a clear competitive relationship between CO_2_ and CH_4_ in the adsorption process by molecular simulation. Long H et al.^25^ found that CO_2_ showed higher adsorption capacity than CH_4_ and N_2_, while Hang L^26^ reported that the adsorption in multi-gas system showed the rule of CO_2_ > CH_4_ > N_2_. In general, these studies confirm that CO_2_ is always dominant in the adsorption of mixed gases.

Although previous studies have made substantial progress, most existing work has mainly focused on the macroscopic analysis of single-gas adsorption or the overall pore characteristics of coal, whereas research on the competitive adsorption behavior of gas power generation tail gas as a specific multicomponent system in coal micropores remains relatively limited. Based on this, the present study, following the pore classification method of Hodot B.B., focuses on the micropore structure of coal (<10 nm) and systematically investigates the competitive adsorption characteristics and pore-size effects of the major components in gas power generation tail gas under micropore conditions. The aim is to reveal the differences in competitive adsorption behavior of multicomponent gases at the microscopic scale and to provide a theoretical basis for CO_2_ storage in goafs and the resource utilization of gas power generation tail gas.

## Materials and methods

### Coal sample preparation

This study takes the Hongqingliang mine in Inner Mongolia, China (for ease of description, marked as HQL below) as an example. In order to ensure the representativeness of the sample and the consistency of the experiment, the coal samples are stored in a sealed bag immediately after collection and brought back to the laboratory for further processing. Firstly, the coal samples were preliminarily screened and broken to the appropriate particle size according to the requirements of pore structure test. Samples with different particle sizes were sorted according to the needs of subsequent experiments. The collection, storage and preparation of all samples are carried out in strict accordance with the national standard GB474–2008 *Preparation method of coal samples*^27^ to ensure the reliability and repeatability of the experimental data.

### Low-temperature nitrogen adsorption (LTGA–N_2_)

Low temperature nitrogen adsorption measurements^28,29^ were conducted on a 3Flex analyzer equipped for determining specific surface area and microporous size distribution. In order to better maintain the inherent pore structure of coal, coal particles in the range of 10–18 mesh particle size are selected. Before the test, the sample was vacuum dried at 110°C for about 6 hours to remove residual moisture and volatile substances. After drying, the sample was loaded into the analytical tube and cooled to liquid nitrogen temperature (77K). During this process, the nitrogen pressure (P/P_0_) was gradually increased to collect the adsorption-desorption isotherms. This experiment was intended to characterize the structural features of the larger micropore range in the coal samples through nitrogen adsorption and to provide experimental data for subsequent molecular simulations.

### Low-pressure carbon dioxide adsorption (LPGA–CO_2_)

Due to the diffusion of nitrogen molecules is restricted in micropores, it is difficult to accurately characterize the ultra-micropores (<2 nm) of the coal samples using nitrogen adsorption alone. To compensate for this limitation, low-pressure carbon dioxide adsorption experiments were further carried out using the 3Flex analyzer^30,31^. After vacuum drying and degassing, the coal samples were placed in the instrument, and the adsorption amount of CO_2_ at different relative pressures (0.0009–0.0299) was measured under an isothermal condition of 273K^32^. This experiment enables an accurate characterization of the structural features of ultra-micropores (<2 nm) in the coal samples and their adsorption capacity for CO_2_.

### Macromolecular model construction and simulation method of coal

**Fig. 1:**
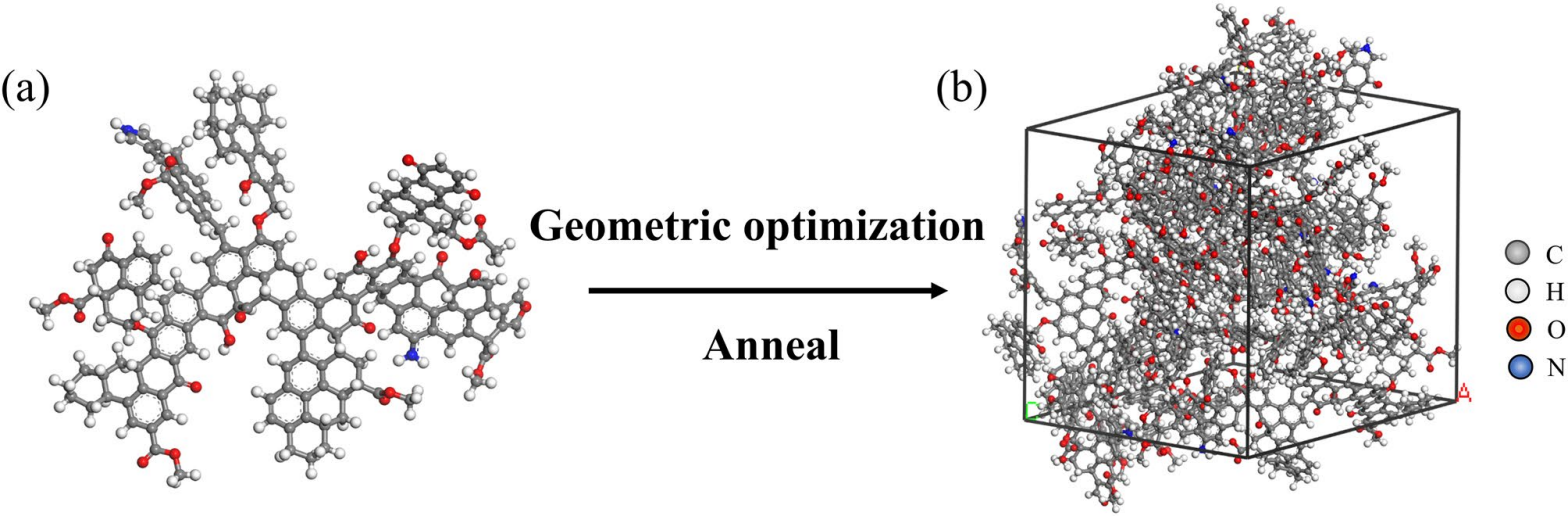
HQL coal molecular model: (**a**) HQL coal amorphous macromolecular structure model; (**b**) Minimum energy structure model of HQL coal.

In the study by Dai et al.^33^, the chemical structure of coal was comprehensively characterized using multiple analytical techniques, and an HQL coal macromolecular model was subsequently constructed. The structural rationality of this model had been validated in previous work by comparison with experimental characterization results, including ^13^C NMR and FT–IR. In the present study, this model was adopted as the basis for simulation. Its molecular formula is C_179_H_138_N_2_O_28_, and the molecular structure is shown in (Fig. 1a). Subsequently, the amorphous molecular cell of HQL coal was developed and balanced using Materials Studio (MS) software. A periodic repetitive framework was generated by Amorph Cell (AC) module. In order to reproduce the intrinsic pore characteristics of coal as realistically as possible, 10 coal molecular units were introduced in each simulation chamber, corresponding to an initial volume density of 1.1g/cm^3^. Then the structure relaxation is carried out through the Forcite module to ensure the energy minimization and geometric stability in front of the balancer.

The COMPASSII force field is used because it provides a broader and more accurate parameterization of the heteroatoms and complex functional groups commonly found in coal macromolecules^34^. In addition, its improved charge distribution algorithm provides more reliable electrostatic and van der Waals interactions, thereby enhancing the physical realism of the simulated structure. After continuous annealing and relaxation steps under the COMPASSII potential, a stable amorphous configuration was obtained. The main optimization parameters are summarized in (Table 1) After optimization, the lowest-energy stable configuration with a density of 1.25g/cm^3^ was obtained. This value is in good agreement with the actual density range of coal (1.2–1.5g/cm^3^), indicating that the model can reasonably reflect the actual structural characteristics of HQL coal. The boundary size of the equilibrium model is 3.554 nm×3.341 nm×3.110 nm, as shown in (Fig. 1b).

**Table 1:** Geometric optimization and annealing optimization parameter settings.

Task	Geometry optimization	Task	Anneal
Quality	Fine	Quality	Fine
Force field	COMPASSII	Force field	COMPASSII
Charge	Forcefield assigned	Electrostatic	Ewald
Electrostatic	Ewald	Van der Waals	Atom based
Van der Waals	Atom based	Initial temperature	300K
Max iterations	5000	Mid-cycle temperature	800K
		Ensemble	NPT

## Pore characteristics of coal and construction of slit-pore model

### Coal pore characteristic

The pore structure of coal has a profound influence on gas adsorption behavior, particularly in the micropore range, where pore size, shape, and distribution directly determine the adsorption capacity of gases^35^. At present, gas adsorption characterization of coal pore structure mainly relies on two methods: low-temperature nitrogen adsorption (LTGA–N_2_) and low-pressure carbon dioxide adsorption (LPGA–CO_2_)^36^.

**Fig. 2:**
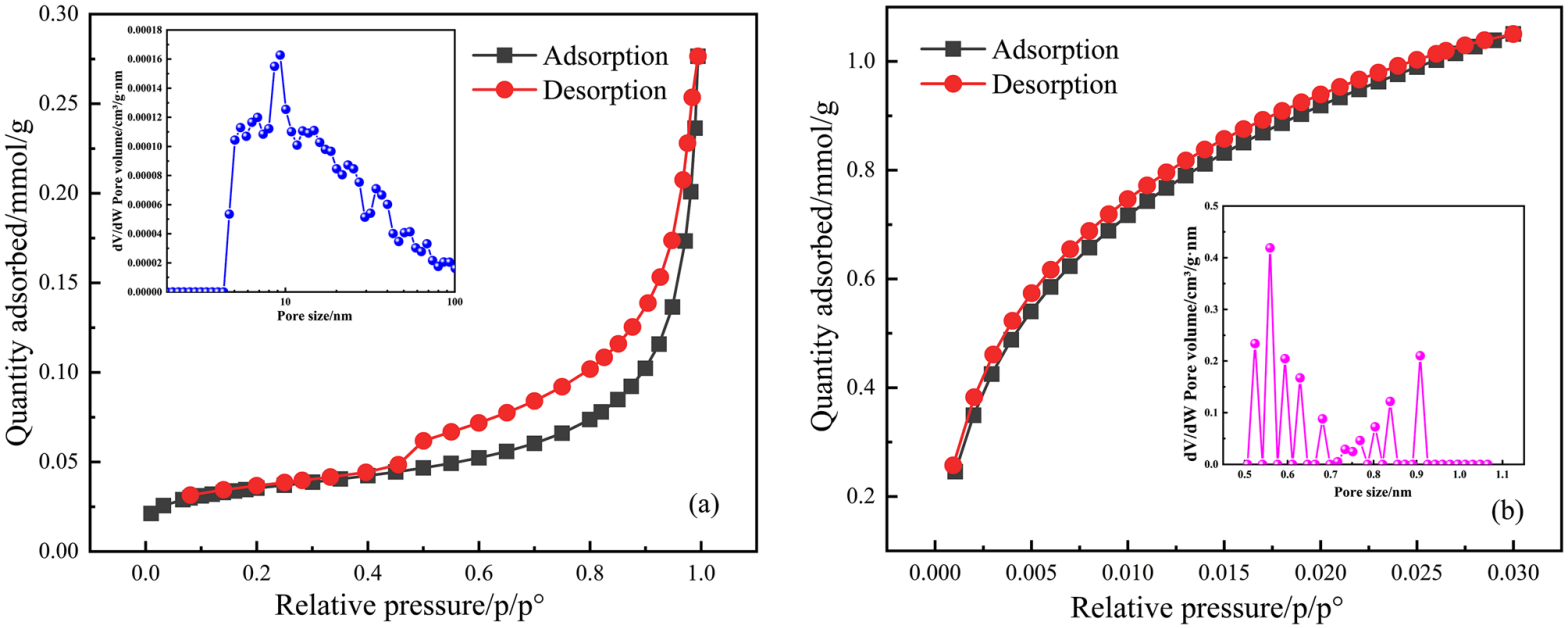
Adsorption isotherm: (**a**) LTGA-N_2_ adsorption/desorption curve; (**b**) LPGA-CO_2_ adsorption/desorption curve.

Owing to the relatively large molecular diameter of N_2_ and its limited diffusivity under low-temperature conditions, LTGA–N_2_ has relatively limited capability for characterizing ultra-micropores and is more suitable for analyzing pores in the range of 2–50 nm. In contrast, CO_2_ molecules can more effectively penetrate ultra-micropores at higher temperatures; therefore, LPGA–CO_2_ is mainly used to characterize ultra-micropore structures smaller than 2nm. It can thus be seen that a single gas adsorption method is insufficient to fully characterize the pore structure of HQL coal below 10 nm. Accordingly, this study combines LTGA–N_2_ and LPGA–CO_2_ to systematically characterize the pore structure of HQL bituminous coal below 10 nm, thereby obtaining a more complete micropore size distribution profile. The experimental results are shown in (Fig. 2).

The LTGA–N_2_ results show that, at low relative pressures, the adsorption amount of the coal sample gradually increases with increasing relative pressure, indicating the presence of abundant open pore structures in the coal. In the relative pressure range of 0.45–0.95, the desorption curve exhibits a clear hysteresis relative to the adsorption curve, forming an H3-type hysteresis loop, which indicates the presence of a large number of slit-shaped pores and a certain proportion of ink-bottle pores in the coal sample^37^. Further pore size distribution analysis based on the DFT model (inset of Fig. 2a) reveals that the pore sizes are mainly distributed in the range of 1.48–216.62 nm and exhibit a distinct multi-peak pattern. Among them, the pore distribution is relatively concentrated in the range of 5.04-11.72 nm, with a peak at 9.31 nm, indicating that pore structures at this scale are relatively well developed.

In the LPGA–CO_2_ experiment, the CO_2_ adsorption/desorption curve (Fig. 2b) rises rapidly at extremely low relative pressures and gradually approaches saturation, indicating the presence of well-developed ultra-micropore structures in the coal sample. The pore size distribution calculated using the DFT model (inset of Fig. 2b) shows that the ultra-micropores are mainly distributed in the range of 0.49–1.07 nm, with a peak at 0.56 nm.

### Construction of slit-pore model

Based on the pore characterization results obtained from LTGA–N_2_ and LPGA–CO_2_ in Section “Coal pore characteristic”, and in accordance with the pore classification method of Hodot B.B. adopted in this study, the present work focuses on the micropore structure of coal below 10nm. The LPGA–CO_2_ results indicate that ultra-micropores in the range of 0.45–1.07 nm are well developed in the coal sample, whereas the LTGA–N_2_ results show that abundant pore structures are present in the range of 1.48–11.72 nm. In addition, the pore size distribution peaks are located at 0.56 and 9.31 nm, respectively. Therefore, to investigate the competitive adsorption behavior of gas power generation tail-gas components in coal micropores under different pore-size conditions, pore widths of 0.5–9nm were selected as the simulation range, and representative pore sizes of 0.5, 0.6, 0.7, 0.8, 1, 2, 3, 5, 7, and 9 nm were employed for simulation. This range can not only capture the main structural characteristics of the HQL coal micropores below 10nm, but also facilitates a systematic comparison of adsorption behavior at different pore scales, thereby ensuring the representativeness and rationality of the simulation results.

**Fig. 3:**
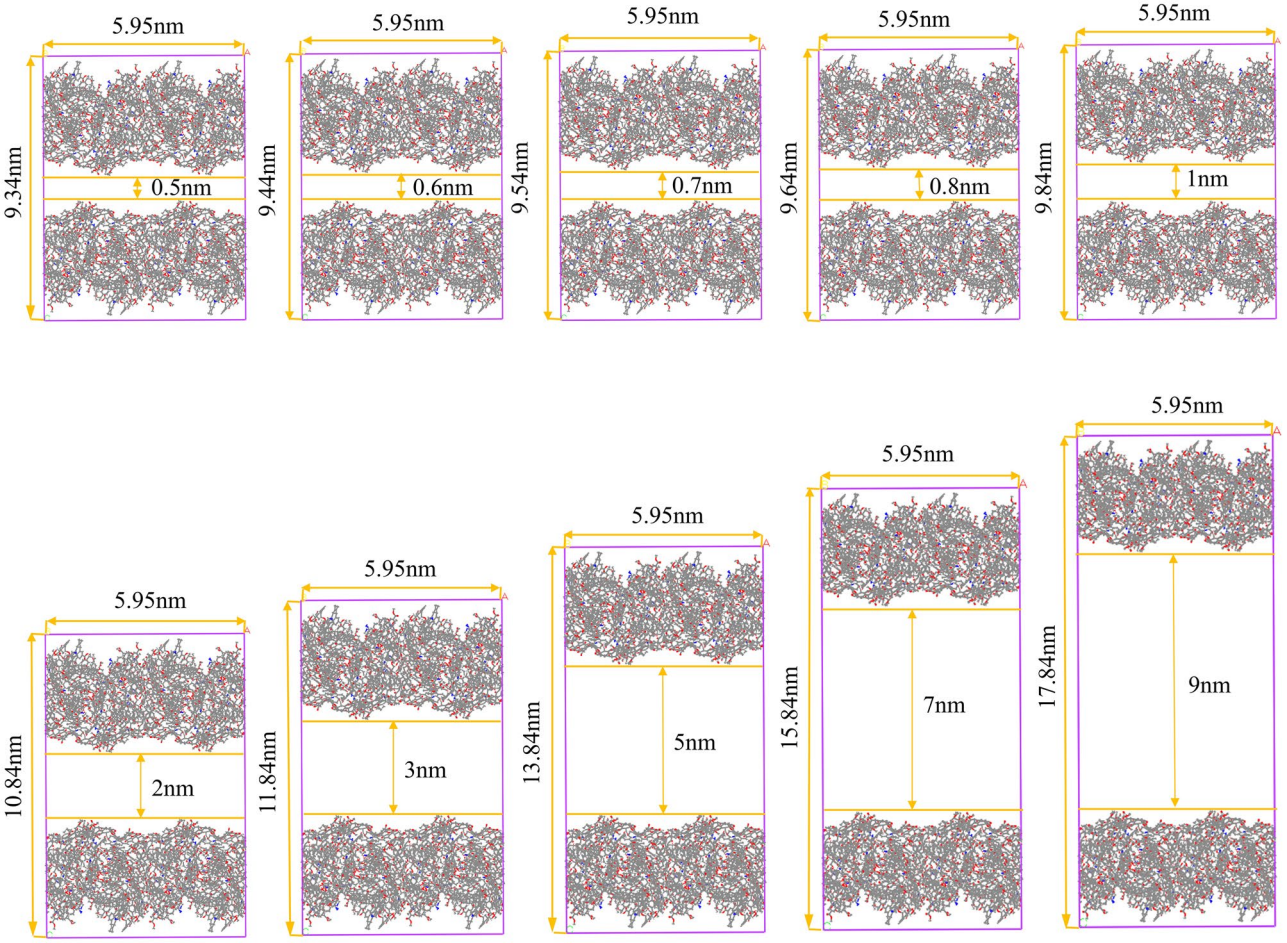
Macromolecular model of coal with different slit pores.

In (Fig. 1b), the coal molecular structure model is double-periodically extended along the x, y and z directions, and a vacuum layer with adjustable thickness is introduced in the z-axis direction to construct a flat slit pore structure with different widths to simulate the typical fracture pore system in the coal seam, as shown in (Fig. 3). Among them, 0.5, 0.6, 0.7, 0.8 and 1nm are defined as ultra-micropore model (<2 nm), the pore sizes of 2, 3, 5, 7 and 9nm are defined as micropore models (> 2nm). which are used to explore the adsorption behavior of gas power generation tail gas at different pore size scales in coal. At the same time, the model structure maintains rigid, symmetrical and periodic boundary conditions. In order to ensure the stability of the structure and the reliability of the subsequent simulation, each pore model was geometrically optimized and annealed in the Forcite module of Materials Studio to obtain the lowest energy configuration for adsorption analysis.

### Simulation parameter setting

In order to study the adsorption characteristics of different gases in the coal pore system, this study used the Sorption module in MS software and combined with the GCMC method. Using this method to simulate the adsorption isotherms of N_2_, CO_2_, O_2_, and gas power generation tail gas. The simulation temperature was set at 298K, which not only facilitates comparison with previous studies but is also close to ambient conditions. The GCMC framework effectively captures the physical adsorption between gas molecules and coal matrix under specified temperature and fugacity conditions, and the simulation results show excellent consistency with the corresponding experimental observations.

In the simulation process, nine discrete pressure points from 0.1 to 8MPa were selected to represent the external environment. Considering that real gases deviate from ideal-gas behavior under medium and high-pressure conditions, fugacity(f), rather than pressure directly, was used to represent the thermodynamic state of the gas during the simulations, so as to reduce the error introduced by the ideal-gas assumption and ensure the thermodynamic consistency of the calculated results. For single-component systems, the external pressure was converted into the fugacity of the corresponding gas for adsorption simulation. For mixed-gas systems, adsorption calculations were carried out under a given total pressure in combination with the partial pressures of each gas component, and the specific gas compositions are listed in (Table 3) Using the Peng-Robinson equation of state^39^, the relationship between pressure and fugacity is determined, as shown in Eq. (1):

1 $$P = \frac{RT}{{V - b}} - \frac{a\left( T \right)}{{V\left( {V + b} \right) + b\left( {V - b} \right)}}$$

Where *P* is pressure, MPa; *T* is temperature, K; *V* is molar volume, m^3^/mol; *R* is the gas constant, 8.314J/(mol·k); *b* is a temperature-independent Van der Waals parameter, and *a* is a temperature-dependent dimensionless function.

When defining the force field parameters, considering the complex non-bond interactions and diverse polar functional groups in the coal molecular skeleton, the COMPASSII force field can be used to accurately describe the adsorption behavior and structural stability. The specific settings are summarized in (Table 2) , where “Atom-based” is used to deal with van der Waals interactions and “Ewald + Group” is used to deal with electrostatic forces. The cut-off distance of the non-bonding interaction is set to 15.5Å. In the simulation process, a 100000balance phase and a subsequent 100,000 sampling phase were implemented to ensure statistical convergence. In order to minimize the uncertainty of the calculation, each data point in this paper represents the average value obtained from three independent simulation replicates.

**Table 2:** Simulation parameter settings.

Setting	Parameter
Forcefield	COMPASSⅡ
Quality	Fine
Electrostatic	Ewald & Group
Van der Waals	Atom based
Ewald accuracy	1.0e−4
Cutoff distance	15.5
Equilibration steps	100000
Production steps	1000000

## Result and discussion

### Adsorption characteristics of CO_2_, N_2_, and O_2_

#### Adsorption isotherm

The exhaust gas after desulfurization and denitrification is mainly composed of about 80%N_2_, 12%CO_2_ and 8%O_2_. In order to explore the effect of pore size on the adsorption characteristics of these main components in coal, GCMC method was used to simulate the single component adsorption of slit pores with 0.59 nm at 298K. The gas composition of each system is shown in (Table 3).

**Table 3:** Gas composition table under different adsorption systems.

Group	Gas composition (%)			
	N_2_	CO_2_	O_2_	He
1	100	–	–	–
2	–	100	–	–
3	–	–	100	–
4	80	12	8	–
5	–	12	–	88
6	80	12	–	8
7	–	12	8	80
8	80	–	–	20
9	80	–	8	12
10	–	–	8	92

The simulated adsorption capacity unit is “molecules/cell”, which is converted to the conventional unit “mmol/g” by Eq. (2)^40^:

2 $$Q = \frac{{N \times \mathop {10}\nolimits^{3} }}{M}$$

In the equation: *Q* is the adsorption amount per unit mass, mmol/g; *N* is the number of gas molecules adsorbed by a unit cell; *M* is the relative molecular mass of the adsorbent, g/mol.

**Fig. 4:**
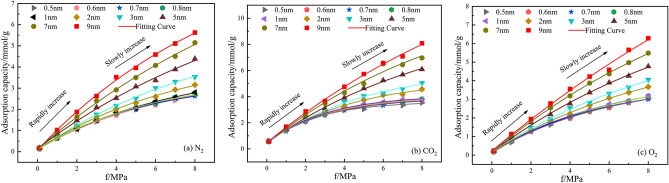
Adsorption isotherms of N_2_/CO_2_/O_2_ in different pore sizes: (**a**) N_2_; (**b**) CO_2_; (**c**) O_2_.

The adsorption behavior of each component is shown in (Fig. 4). At low fugacity (f<2MPa), the adsorption capacity of all gases increased sharply, reflecting the strong initial filling of micropores. With the increase of fugacity, the growth rate of adsorption capacity gradually slowed down. After more than 5MPa, the adsorption capacity was almost unchanged, indicating that the micropore site was saturated and the Langmuir type behavior began to appear^41^. In this region, the isotherm slope of the ultra-micropores is smoother, showing a weaker pressure dependence.

For the 1nm pore system, the equilibrium adsorption capacities of N_2_, CO_2_ and O_2_ are 1.07, 2.12 and 1.28mmol/g at f = 2MPa, respectively. When the pore size increased to 9nm, the corresponding adsorption capacity increased to 0.81, 0.75 and 0.66mmol/g, respectively. This indicates that the adsorption capacity usually increases with the expansion of the pore size, which is attributed to the additional accessible surface area. Under the same conditions, the average adsorption capacity of CO_2_ is about 2 times that of N_2_ and about 1.7 times that of O_2_, indicating that it has a stronger affinity for coal matrix.

At medium pressure (f = 5MPa), the adsorption capacity of CO_2_ is about 1.7 times that of N_2_ and about 1.4 times that of O_2_. At high pressure (f = 8MPa), the adsorption capacity of CO_2_ is only 1.4 times that of N_2_ and about 1.3 times that of O_2_. This indicates that the pressure change significantly affects the relative adsorption amount between gases. As the saturation approaches, the ability difference between gases gradually decreases. However, CO_2_ shows the highest adsorption capacity at all test pressures, indicating that the interaction between CO_2_ and coal surface is still much stronger than that between N_2_ or O_2_ at the same temperature and pressure.

#### Adsorption heat

The isosteric heat of adsorption reflects the strength of gas-solid interactions and is therefore a sensitive indicator of affinity between the coal surface and gas molecules. It can be calculated in MD using the wave theory at constant temperature Eq. (3). See Ref.^42^ for details.

3 $$\mathop q\nolimits_{st,T} = \frac{{\left\langle {\mathop N\nolimits_{b} } \right\rangle }}{{f\left\langle {\mathop N\nolimits_{b} ,\mathop N\nolimits_{b} } \right\rangle }}\mathop \kappa \nolimits_{B} T - \frac{{f\left( {U,N} \right)}}{{f\left( {U,N} \right)}}$$

In the formula, *f* <*N*_*b*_, *N*_*b*_>=<*N*_*b*_, *N*_*b*_>-<*N*_*b*_><*N*_*b*_> is the fluctuation variable, which is the ensemble mean of the variable <*N*_*b*_>. *N*_*b*_ is the number of gas molecules in volume *V*_*b*_ affected by the same chemical potential as the loading system. *К*_*B*_ is a Boltzmann constant. *U* and *N* are the configuration energy and the number of molecules of the system, respectively.

**Fig. 5:**
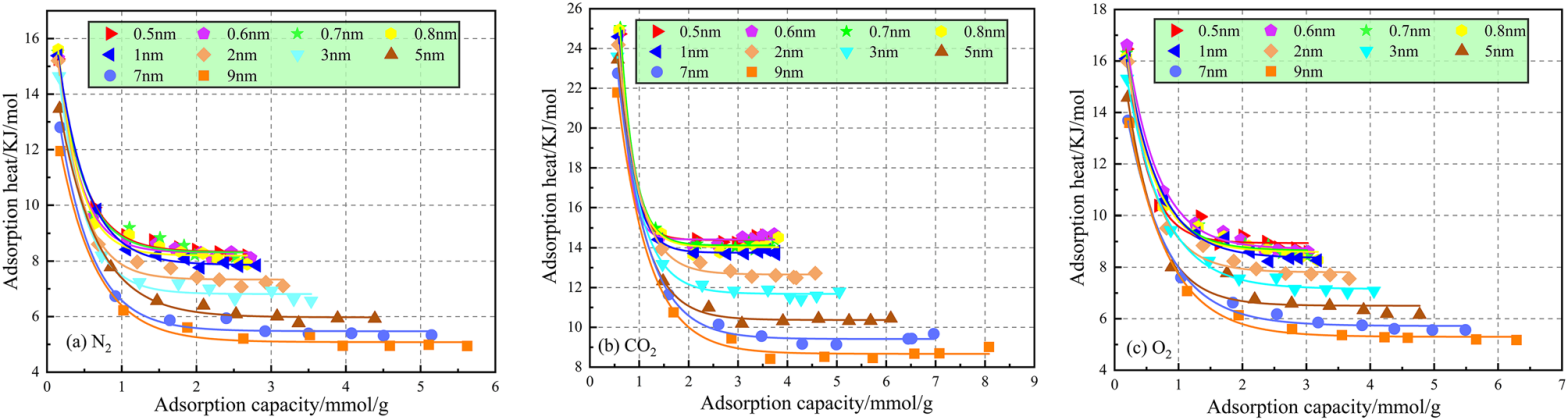
Relationship between adsorption heat and adsorption capacity.

Figure 5 compares the adsorption heat of each gas component of the gas power generation tail gas in the micropore. In the low adsorption capacity stage (<1mmol/g), the initial adsorption heat reaches the highest, indicating that each gas preferentially occupies the high-energy adsorption site on the surface of the coal pore wall at the initial stage of adsorption. With the increase of adsorption capacity, the adsorption heat of each gas showed an exponential downward trend, which showed a rapid decline and then tended to be gentle. This indicates that as the high-energy sites are gradually occupied, the subsequent gas molecules are more distributed at the adsorption sites with lower energy.

In addition, it can be seen that the heat released by N_2_, CO_2_ and O_2_ in ultra-micropores is significantly higher than that in micropores. The average adsorption heat in ultra-micropores is about 9.27,15.39 and 9.86kJ/mol, respectively, while the average adsorption heat in larger micropores is about 20% lower than that in ultra-micropores. This shows that the decrease of pore size significantly enhances the interaction between gas molecules and coal pore wall. In the ultra-micropores, due to the narrow channel, the potential field of the pore wall is superimposed, and the gas molecules are attracted by the pore walls on both sides at the same time, thus showing higher adsorption heat. With the increase of pore size, the superposition effect of pore wall potential field is weakened, and the interaction between gas and coal decreases.

A comparison among the different gas components shows that the heat of adsorption of CO_2_ is consistently higher than that of N_2_ and O_2_, following the overall order CO_2_ > O_2_ > N_2_. This is mainly because CO_2_ possesses a larger quadrupole moment and higher polarizability, enabling it to interact more strongly with the coal matrix in the heterogeneous potential field on the pore surface. As a result, CO_2_ exhibits a higher heat of adsorption and stronger adsorption affinity. In contrast, the intermolecular interactions of O_2_ and N_2_ are relatively weaker, with N_2_ showing the weakest adsorption. This difference is particularly amplified under ultra-micropore conditions, where the energetic disparity among gas molecules becomes more pronounced, making the adsorption advantage of CO_2_ even more evident. This thermodynamic behavior is completely consistent with the isotherm results in Section “Adsorption isotherm”, and further explains the reason why CO_2_ has the strongest competitive advantage in coal-adsorbed gas mixtures.

### Competitive adsorption mechanism of gas power generation tail gas

#### Competitive adsorption capacity analysis

**Fig. 6:**
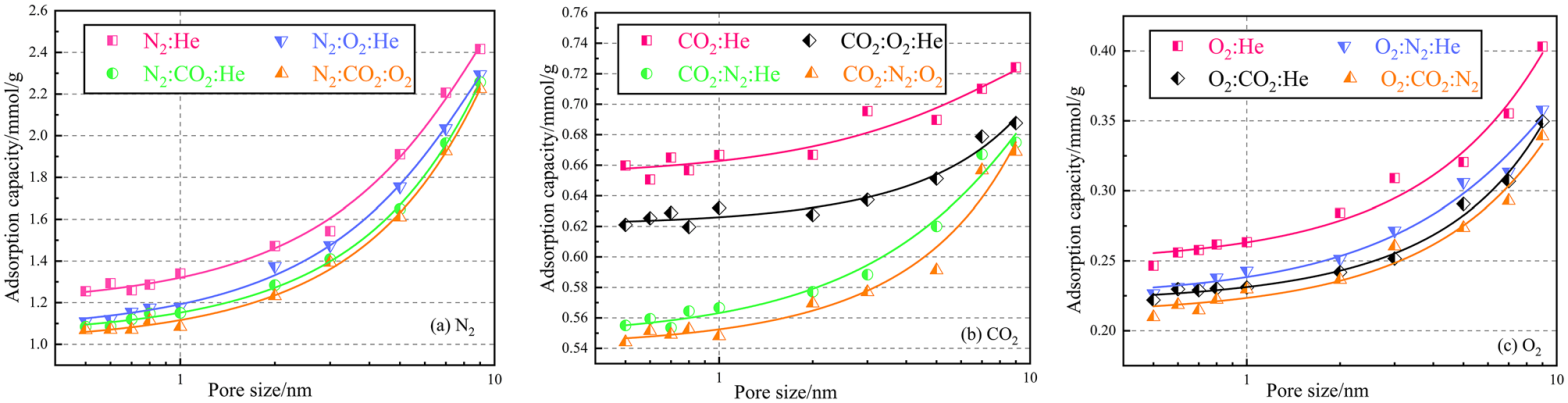
Competitive adsorption capacity of gas power generation tail gas (**a**–**c**) are the adsorption capacity of N_2_, CO_2_ and O_2_ in the mixed gas, respectively).

In this section, the competitive adsorption behavior of N_2_, CO_2_ and O_2_ in the power generation exhaust was analyzed under the actual emission components^43^. Three simulation environments were established: one-dimensional, two-dimensional and three-dimensional gas systems. In each case, helium is introduced as an inert equilibrium gas to maintain the same total pressure in all devices. The detailed composition of the simulated gases is shown in (Table 3), and the adsorption results are shown in (Fig. 6).

Figure 6a shows the response of N_2_ adsorption under different mixing conditions. In the monocomponent system (N_2_﹕He), the adsorption capacity of N_2_ reached 1.6mmol/g. When CO_2_ or O_2_ was added, the adsorption capacity of N_2_ decreased by 8.14 and 11.39%, respectively, due to the stronger affinity of these competitive molecules to the coal surface. At the same time, after the introduction of CO_2_ and O_2_, the reduction of N_2_ adsorption amount further reached 13.64%. The behavior of CO_2_ and O_2_ follows a similar trend, as shown in Fig. 6b and c. In the CO_2_﹕He system (Fig. 6b), the addition of N_2_, O_2_ and their mixture reduced the amount of CO_2_ adsorption by 5.46, 12.54 and 14.31%, respectively. Notably, after O_2_ was introduced into the binary adsorption system, the adsorption amount of CO_2_ decreased by 2.02%. This indicates that the introduction of O_2_ exerts a certain competitive effect on CO_2_ adsorption, as some adsorption sites are occupied by O_2_, leading to a slight decrease in the adsorption amount of CO_2_. This suggests that the presence of O_2_ weakens the adsorption and sequestration of CO_2_ to some extent. In the O_2_﹕He system (Fig. 6c), after the introduction of N_2_, CO_2_, and their mixed gas, the average adsorption amount of O_2_ decreased by 0.98, 12.84, and 15.54%, respectively.

In general, the overall adsorption capacity of the three gases has the same trend: adding another or two mixed gases to the one-component system will reduce the adsorption capacity of the gas. In the process of gas adsorption, the inhibition effect in the ultrafine pore structure is obviously greater than that in the larger micropore. This indicates that the gas molecules are more competitive for the occupation of high-energy adsorption sites in the ultra-microporous structure, which is significantly weakened in the larger micropore range; after increasing from the single system to the multi-component adsorption system atmosphere, the competitive effect between the components gradually increased.

**Fig. 7:**
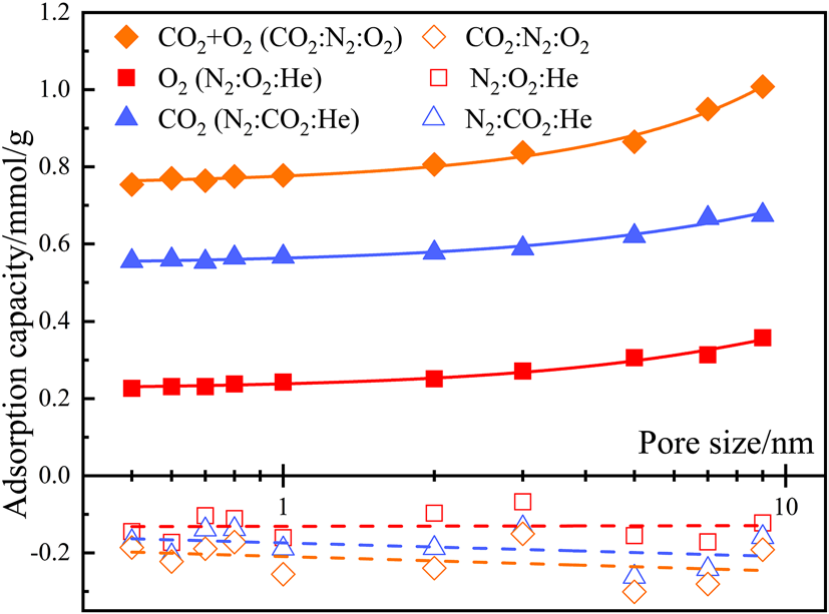
Competitive adsorption capacity (solid line is the adsorption capacity of other gases except N_2_ and He in the mixture; the imaginary line is the difference between the adsorption amount of N_2_ in each mixed gas and the adsorption amount of N_2_ in the unary system (N_2_﹕He).

Figure 7 further illustrates the relationship between the decrease of N_2_ adsorption amount and the evolution of adsorption capacity as the gas composition transitions from one component to a mixed system represented by power generation tail gas. When O_2_ (N_2_﹕O_2_﹕He) was added to the N_2_﹕He system, the adsorption amount of N_2_ decreased by 0.13mmol/g, while the adsorption amount of O_2_ was 0.27mmol/g. After adding CO_2_ (N_2_﹕CO_2_﹕He), the adsorption capacity of N_2_ decreased by 0.18mmol/g, and the adsorption capacity of CO_2_ reached 0.59 mmol/g. When O_2_ and CO_2_ (N_2_﹕O_2_﹕CO_2_) were added at the same time, the adsorption capacity of N_2_ was further reduced by 0.22 mmol/g, and the total adsorption capacity of the two gases reached 0.83 mmol/g.

Compared with pure N_2_ atmosphere, the adsorption capacity of N_2_ on coal decreases with the introduction of external gas. The order of N_2_ adsorption capacity is N_2_﹕O_2_﹕CO_2_ < N_2_﹕CO_2_﹕He < N_2_﹕O_2_﹕He < N_2_﹕He, indicating that the presence of CO_2_ has the strongest competitive inhibition. At the same time, since the newly introduced gas occupies the high-energy sites vacated by the N_2_ molecule, the total absorption of all gases increases. Therefore, the competitive ability of CO_2_ and O_2_ is significantly greater than that of N_2_, which is consistent with the higher adsorption heat and molecular polarity discussed in Section “Adsorption characteristics of CO_2_, N_2_, and O_2_”. CO_2_ molecules preferentially occupy the most active adsorption sites, and the overall competition order is CO_2_ > O_2_ > N_2_.

**Fig. 8:**
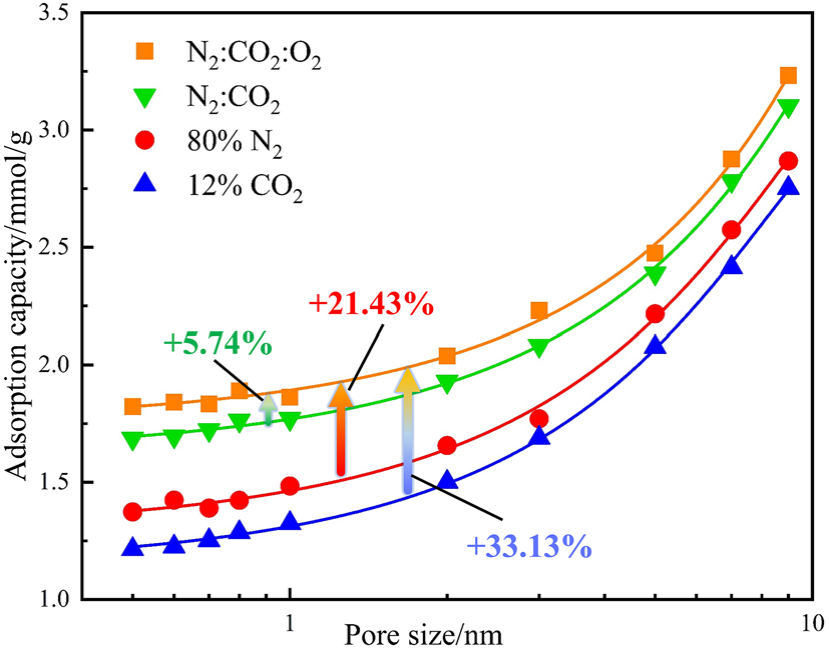
Total gas adsorption.

Figure 8 shows the change of total adsorption capacity of coal samples under different atmosphere conditions. It can be seen that the total adsorption capacity in the mixed atmosphere of gas power generation tail gas is higher than that in other atmosphere systems in the whole pore size range. Compared with N_2_: CO_2_ system, the total adsorption capacity increased by about 5.74%. Compared with the N_2_-based atmosphere system (80%N_2_), the total adsorption capacity increased by about 21.43%. Compared with 12%CO_2_ atmosphere, the total adsorption capacity can be increased by 33.13%. These results indicate that both CO_2_ and O_2_ in the gas power generation tail-gas mixture contribute to the enhancement of the total adsorption capacity of the system, with CO_2_ making the most significant contribution. It should be noted that (Fig. 6b) reflects the competitive inhibitory effect of O_2_ on the adsorption of the CO_2_ component, namely, the addition of O_2_ leads to a slight decrease in CO_2_ adsorption (about 2.02%); by contrast, the present result reflects the change in the total adsorption capacity of the entire multicomponent system. Although O_2_ slightly suppresses the adsorption of CO_2_, O_2_ itself can still participate in adsorption and occupy pore space. Therefore, from the overall system perspective, the total adsorption capacity of the tail-gas mixture still increases.

#### Adsorption selectivity

The adsorption behavior of multi-component gases is determined by the relative affinity of each component to the coal surface, which can be quantitatively described by the selectivity coefficient^44^, defined by Equation (4)^45^:

4 $$\mathop S\nolimits_{{{i \mathord{\left/ {\vphantom {i j}} \right. \kern-0pt} j}}} = \frac{{{{\mathop x\nolimits_{i} } \mathord{\left/ {\vphantom {{\mathop x\nolimits_{i} } {\mathop x\nolimits_{j} }}} \right. \kern-0pt} {\mathop x\nolimits_{j} }}}}{{{{\mathop y\nolimits_{i} } \mathord{\left/ {\vphantom {{\mathop y\nolimits_{i} } {\mathop y\nolimits_{j} }}} \right. \kern-0pt} {\mathop y\nolimits_{j} }}}}$$

**Fig. 9:**
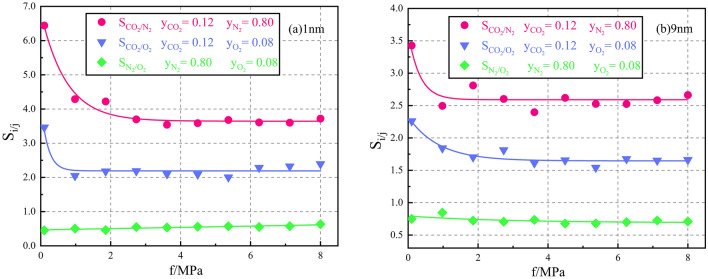
Gas selectivity coefficient in binary adsorption system under different pore sizes: (**a**) 1nm; (**b**) 9nm.

**Fig. 10:**
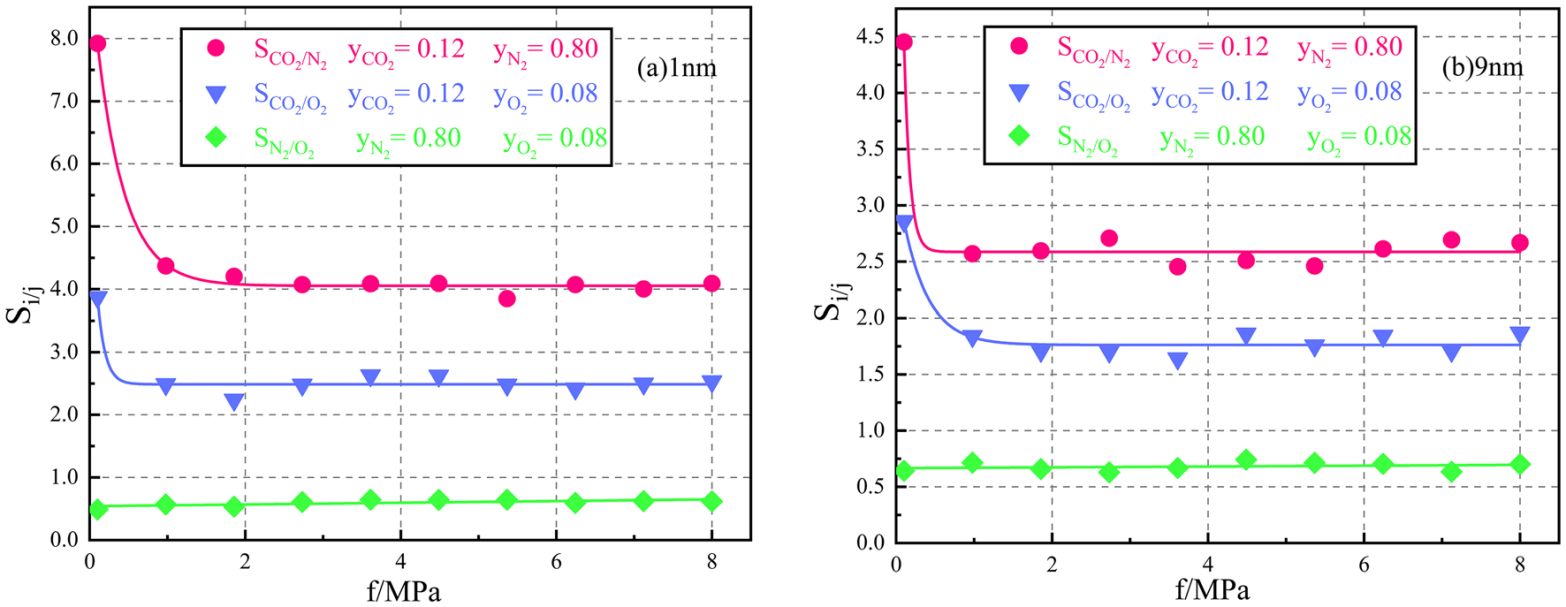
Gas selectivity coefficient in ternary adsorption system under different pore sizes: (**a**) 1nm; (**b**) 9nm.

In the formula: *x*_*i*_ and *x*_*j*_ represent the mole fraction of components i and j in the adsorption phase, respectively, and *y*_*i*_ and *y*_*i*_ represent the mole fraction of components i and j in the gas phase, respectively. In the gas adsorption system, when *S*_*i*/*j*_ >1, component i shows stronger adsorption than component j. To facilitate comparison of the competitive adsorption characteristics at different pore scales, 1nm and 9nm were selected as representative pore sizes for ultra-micropores and larger micropores, respectively. The adsorption selectivity coefficients of the binary adsorption systems and gas power generation tail gas in coal molecules are shown in (Figs. 9 and 10), respectively.

In the binary adsorption system with 1nm pore structure (Fig. 9a), the initial selectivity (*S*_*CO2*/*N2*_) of CO_2_ to N_2_ reached 6.4, which decreased sharply with the increase of pressure and stabilized at about 3.6 at f = 4. For *S*_*CO2*/*O2*_, the corresponding coefficient starts at 3.5 and tends to be stable around 2.1, while *S*_*N2*/*O2*_ remains almost constant at about 0.5, showing the minimum sensitivity to pressure. In the 9nm pore system (Fig. 9b), *S*_*CO2*/*N2*_ was initially 3.5, gradually approaching 2.6 with increasing pressure, while *S*_*CO2*/*O2*_ decreased from 2.3 to 1.6, and *S*_*N2*/*O2*_ remained around 0.7. In all cases, the selectivity of CO_2_ to N_2_ and O_2_ is always >1, indicating that CO_2_ consistently dominates the competitive adsorption process. By contrast, *S*_*N2*/*O2*_ is always <1, indicating that N_2_ is always in a weak adsorption state in the binary system. Compared with the 1nm pore, both *S*_*CO2*/*N2*_ and *S*_*CO2*/*O2*_ at 9nm are markedly lower, indicating that the competitive ability of CO_2_ for adsorption sites weakens as pore size increases, and the competitive effect is significantly reduced. Therefore, the ultra-micropore structure is more favorable for amplifying the selectivity advantage of CO_2_ over N_2_ and O_2_.

The variation trend of the selectivity coefficient in the tail gas of gas power generation (Fig. 10) is similar to that of the binary system, but the value increases slightly. At a pore size of 1nm (Fig. 10a), *S*_*CO2*/*N2*_ reaches 7.9 in the low-pressure region (<2MPa), decreases rapidly with increasing pressure and stabilizes at 4.0 in the high-pressure region (>5MPa). *S*_*CO2*/*O2*_ decreases from 4.0 to 2.5, while *S*_*N2*/*O2*_ remains at 0.5, which is the same as the coefficient in the binary mixed system (Fig. 9a). When the pore structure increases to 9nm, *S*_*CO2*/*N2*_ decreases from 4.5 to 2.6, *S*_*CO2*/*O2*_ decreases from 2.9 to 1.7, and *S*_*N2*/*O2*_ is the same as the coefficient of (Fig. 9b). These results indicate that under the atmosphere of gas power generation tail gas, the competitive advantage of CO_2_ still exists, and this advantage is more pronounced under ultra-micropore and low-pressure conditions.

Although the selectivity coefficient values are different under different systems and pore size conditions, the overall law is consistent, that is, the strong adsorption component CO_2_ has the most significant competitive advantage under low pressure and ultra-micropore conditions. As the pressure increases or the pore size increases, the selectivity coefficient gradually decreases and tends to be stable. This indicates that the micropores of coal, especially the ultra-micropore structure, have obvious preferential adsorption and enrichment ability for CO_2_. In engineering applications, this feature means that after the tail gas of gas power generation is injected into the goaf, CO_2_ is more likely to be preferentially retained in the micropores of the coal body, while N_2_ and O_2_ are relatively more difficult to be preferentially adsorbed, which is conducive to improving the storage efficiency of CO_2_ and the utilization level of tail gas resources.

### Influence of pore size on competitive adsorption characteristics

In this section, combined with the analysis results of 4.1 and 4.2, the microscopic mechanism of coal adsorption on gas power generation tail gas is further revealed in the micropore range.

**Fig. 11:**
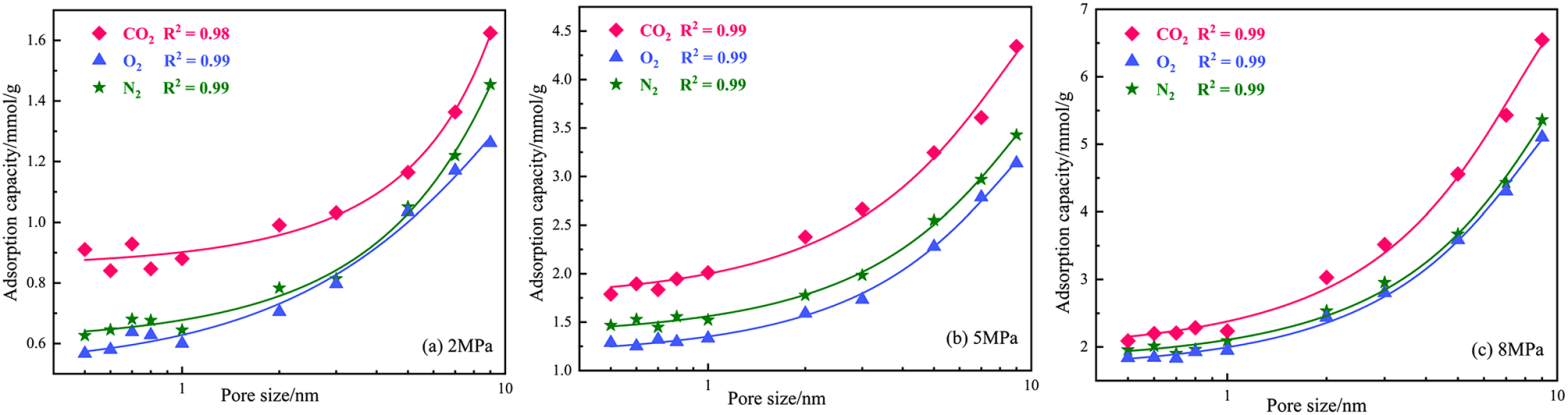
The adsorption capacity of N_2_/CO_2_/O_2_ slit pores under different pressures: (**a**) 2MPa; (**b**) 5MPa; (**c**) 8MPa.

Due to the high irregularity of the surface of coal molecules, it is difficult to accurately define the boundary between the slit pore area and the original pore structure at the atomic scale after the vacuum layer is introduced. If the adsorption amounts of slit pores are calculated directly based on the local distribution integral, the results will inevitably be affected by the uncertainty of the boundary definition. To minimize the uncertainty arising from model boundary effects, a difference approach was applied, in which the additional contribution of slit pores was quantified by comparing the adsorption capacity between slit-pore and non-slit-pore models under identical conditions. This method reasonably isolates the influence of slit geometry while maintaining the intrinsic structural integrity of the coal matrix. Based on the adsorption data of (Fig. 4), the adsorption capacity of each gas in different slit pores under different pressures was calculated. In order to facilitate the comparison of adsorption differences under different pressures, 2, 5 and 8MPa were selected as representative pressure conditions, corresponding to low pressure, medium pressure and high pressure, respectively. The results are shown in (Fig. 11).

At 2MPa (Fig. 11a), the adsorption capacity of the three gases increased with the increase of pore size. In the ultra-microporous region (<2nm), the growth rate of CO_2_ adsorption is particularly steep, reflecting its stronger intermolecular interaction and space filling ability in narrow pores. When the pressure increases to 5MPa (Fig. 11b), the total adsorption capacity further increases, especially in the micropore range (2–9nm), and the slope of the isotherm becomes steeper. Under this condition, CO_2_ maintains a leading adsorption capacity in the entire pore. Although N_2_ and O_2_ also show increased adsorption at larger pore sizes, the rate of change is still relatively flat, reflecting a weak interaction with the pore wall. At 8MPa (Fig. 11c), the micropore and ultra-micropore area curves showed a clear upward trend, confirming that the pressure enhanced the role of pore size in adsorption. The adsorption of CO_2_ in the pores below 2nm is close to saturation, but the adsorption capacity continues to increase in the range of 2–9 nm, while N_2_ and O_2_ show smaller and more gentle changes.

In general, the increase of pressure and pore width significantly enhanced the adsorption of all gases, and the enhancement of CO_2_ was the most significant. This observation emphasizes the key role of slit-shaped micropores and ultra-micropores in determining the preferential adsorption of CO_2_ and the competitive behavior between flue gas components.

**Fig. 12:**
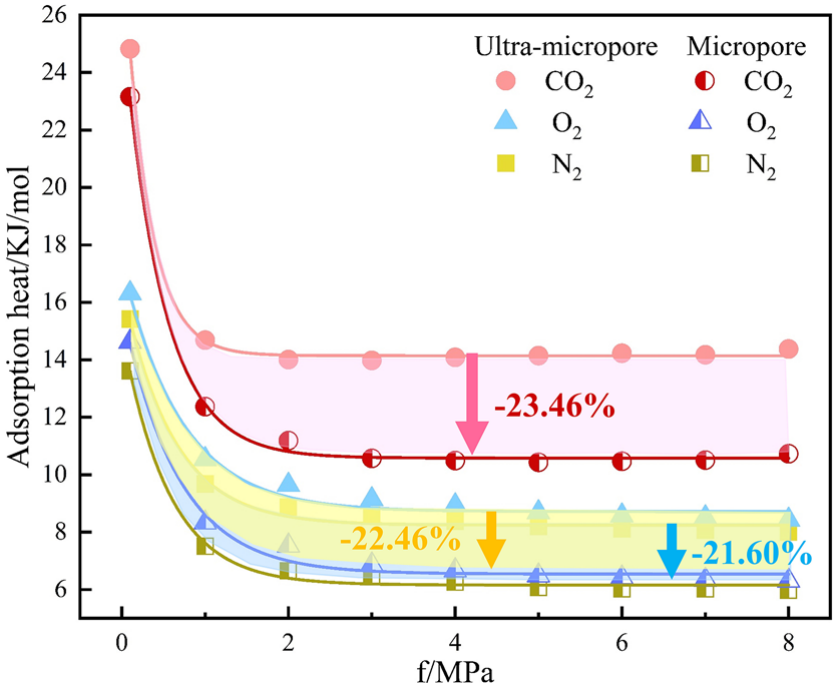
Difference of adsorption heat in different micropores.

Similarly, according to (Fig. 5), the average adsorption heats of each gas in ultra-micropores and micropores were compared (Fig. 12). The corresponding values of CO_2_, O_2_ and N_2_ are 15.39, 9.86 and 9.26 kJ/mol, respectively. When the pore size expanded from ultra-micropores to micropores, the average adsorption heat decreased by 23.46, 22.46 and 21.60%, respectively. This indicates that the gas-solid interaction energy is more concentrated in the narrower pores, especially CO_2_. The average adsorption heat in the micropores is higher than that in the micropores, and CO_2_ always shows the strongest adsorption enthalpy in the three gases. These findings are consistent with section “Adsorption heat”. This indicates that although the ultra-micropores are at a disadvantage in capacity, they significantly amplify the affinity difference between different gas molecules at the energy level, revealing that CO_2_ has a more obvious adsorption driving force due to its polarity and charge distribution.

**Fig. 13:**
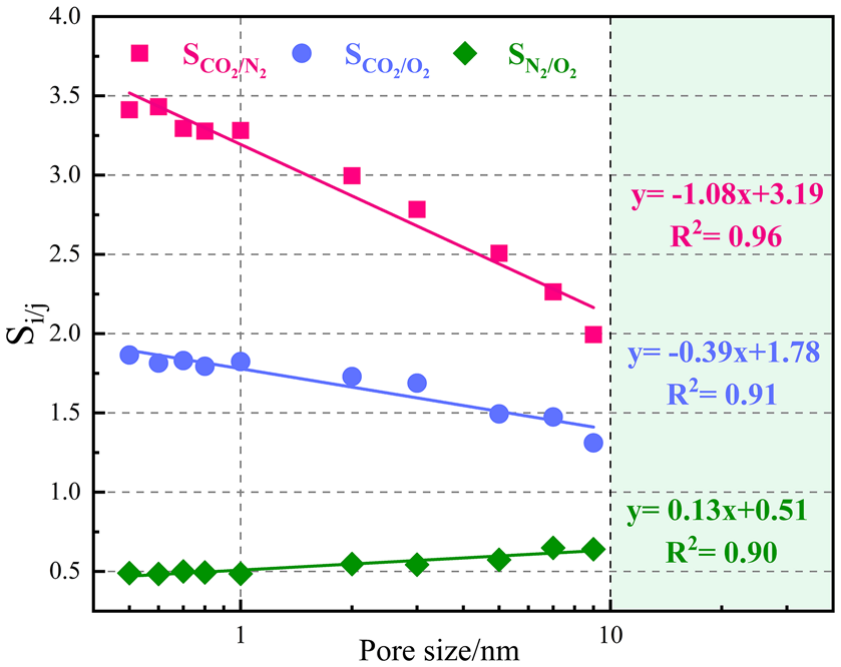
Adsorption selectivity of mixed gas varies with pore size.

In addition, from the results of competitive selectivity (Fig. 13), it can be seen that the selectivity of *S*_*CO2*/*N2*_ and *S*_*CO2*/*O2*_ decreased with the increase of pore size, reaching 3.5 and 1.8 in ultra-micropores, respectively, but decreased to about 2.0 and 1.4 in micropores, indicating that ultra-micropores played a prominent role in amplifying the adsorption difference between CO_2_ and other gases, and the increase of pore size weakened this difference. In contrast, the selectivity of *S*_*N2*/*O2*_ is always lower than 1, and the overall change range is limited, indicating that the affinity of the two in the coal matrix is close, and it is difficult to achieve effective separation.

In summary, the microporous structure of coal shows a “capacity-energy-selectivity” synergistic mechanism in the adsorption of tail gas from gas power generation: micropores determine the scale of adsorption capacity, and ultra-micropores enhance energy difference and gas selectivity. CO_2_ maintains a dominant position in the competition due to its higher adsorption heat and significant micropore advantages. This mechanism reveals the division and cooperation of multi-scale pore size in the regulation of capacity and selectivity, and provides a theoretical basis for the efficient utilization of tail gas from gas power generation and the preferential capture of CO_2_.

## Conclusions

1. In the single-component system, the adsorption order of the three gases follows CO_2_ > O_2_ > N_2_. The average adsorption capacity of CO_2_ is 2.12 mmol/g, which is about 1.5 times that of O_2_ and 1.7 times that of N_2_. The adsorption heat of CO_2_ is 15.39 kJ/mol, which is higher than that of O_2_ (9.86 kJ/mol) and N_2_ (9.26 kJ/mol), indicating that it has stronger interaction with coal matrix and higher energy affinity.
2. In the process of multi-component competitive adsorption of gas power generation tail gas, the order of gas competition is still as follows: CO_2_ > O_2_ > N_2_. CO_2_ is always dominant, and the presence of O_2_ has a certain inhibitory effect on CO_2_ adsorption (about 2.02%), but this effect is mainly reflected in the component competition level; from the overall system, the synergistic effect between the exhaust gas components increased the total adsorption capacity by 21.43% compared with the pure N_2_ atmosphere. At the same time, CO_2_ shows a more significant selective advantage in the ultra-microporous range.
3. The change of coal pore size has a significant regulatory effect on gas adsorption behavior. The supermicropores (<2 nm) structure not only enhances the interaction between gas molecules and coal, but also significantly amplifies the competition and energy difference between different gases. *S*_*CO2*/*N2*_ was as high as 3.5 and *S*_*CO2*/*O2*_ was about 1.8. When the pore size increases to larger micropores, the two decrease to 2.0 and 1.4, respectively, indicating that the competitive advantage of CO_2_ decreases with the increase of pore size. As the pore size increases from 0.5 to 9 nm, the adsorption capacity of the three gases increases, but the average adsorption heat decreases by about 20%, indicating that the energy contribution of ultra-micropores plays the most critical role in the adsorption behavior.

## Data Availability

Data will be made available on request.
